# Fruit and Vegetable Consumption and Changes in Anthropometric Variables in Adult Populations: A Systematic Review and Meta-Analysis of Prospective Cohort Studies

**DOI:** 10.1371/journal.pone.0140846

**Published:** 2015-10-16

**Authors:** Lukas Schwingshackl, Georg Hoffmann, Tamara Kalle-Uhlmann, Maria Arregui, Brian Buijsse, Heiner Boeing

**Affiliations:** 1 German Institute of Human Nutrition, Arthur-Scheunert-Allee 114–116, 14558 Nuthetal, Germany; 2 Department of Nutritional Sciences, Faculty of Life Sciences, University of Vienna, Althanstraße 14 (UZAII), A-1090 Vienna, Austria; St Francis Hospital, UNITED STATES

## Abstract

**Background:**

Randomized controlled trials provide conflicting results on the effects of increased fruit and vegetable consumption on changes in body weight. We aimed to perform a systematic review and meta-analysis of prospective cohort studies on fruit and vegetable consumption in relation to changes in anthropometric measures.

**Methods:**

PubMed and EMBASE were searched up to July 2015 for prospective studies reporting on habitual fruit and/or vegetable consumption in relation to changes in body weight or waist circumference or to risk of weight gain/overweight/obesity in adults. Random-effects meta-analysis was applied to pool results across studies.

**Findings:**

Seventeen cohort studies (from 20 reports) including 563,277 participants met our inclusion criteria. Higher intake of fruits was inversely associated with weight change (decrease) (beta-coefficient per 100-g increment, -13.68 g/year; 95% CI, -22.97 to -4.40). No significant changes could be observed for combined fruit and vegetable consumption or vegetable consumption. Increased intake of fruits was inversely associated with changes (decrease) in waist circumference (beta: -0.04 cm/year; 95% CI, -0.05 to -0.02). Comparing the highest combined fruit & vegetable, fruit, and vegetable intake categories were associated with a 9%, 17%, and 17% reduced risk of adiposity (odds ratio [OR]: 0.91, 95% CI, 0.84 to 0.99), (OR: 0.83, 95% CI, 0.71 to 0.99), and (OR: 0.83, 95% CI, 0.70 to 0.99), respectively.

**Conclusion:**

This meta-analysis showed several inverse associations between fruit and vegetable intake and prospective improvements in anthropometric parameters, and risk of adiposity. The present meta-analysis seems to be limited by low study quality. Nevertheless, when combined with evolutionary nutrition and epidemiological modeling studies, these findings have public health relevance and support all initiatives to increase fruit and vegetable intake.

## Introduction

International and national dietary guidelines recommend an increased intake of fruit and vegetables. The World Health Organization and the Food and Agriculture Organization, for example, recommend to eat at least 400 g of fruit and vegetables per day, excluding potatoes and other tuberous root vegetables [1]. Several national authorities launched “5-a-day” campaigns with recommendations to eat up to 650 g fruit and vegetables per day [2].

The recommendation to eat a certain amount of fruit and vegetables is based on evaluation of short-term randomized controlled trials on cardiovascular risk factors, associated with blood pressure reductions [3–5] and of long-term prospective observational studies from which the majority report that a higher fruit and vegetable intake is associated with a reduced risk of all-cause mortality, cardiovascular disease, diabetes, hypertension and several cancer types [2, 6].

As importantly, and as should be excepted, studies [7, 8] of human evolutionary (or ancestral) nutrition, and basic and antioxidant science, gives strong evidence for types of mechanisms involved in plant food conferring wide ranging with metabolic health [9–11].

One of the additional impacts of a diet consisting of a high proportion of fruit and vegetables aside from chronic diseases could be a slower weight gain over time compared to diets with a low proportion. Fruit and especially non-starchy vegetables have a low energy density and thus may help in preventing weight gain [12]. However, the second World Cancer Research Fund/American Institute for Cancer Research (WCRF/AICR) Expert Report from 2007 concluded that there was only probable evidence for an association between low energy-dense foods including non-starchy vegetables and a decreased risk of weight gain, overweight, and obesity [13]. For fruit the evidence was considered as limited and inconclusive [13]. A more recent critical evaluation of the evidence from prospective studies from 2012 by a different group from WCRF concluded that there is possible evidence that an increase in the consumption of fruit and vegetables contributes to weight stability [2].

However, when considering studies in this research field one is faced with different constructs. Whereas in one type of study habitual diet is investigated with respect to its long-term effect on weight, in the other type of study, change in fruit and vegetable consumption on weight change is investigated.

Two recent meta-analyses of randomized trials (RCTs) on change in dietary fruit and vegetable and changes in body weight gave conflicting results. The first meta-analysis included eight trials and reported that an increase in fruit and vegetable intake (on average 133g) induced a 0.68 kg smaller body weight increase during a median duration of 14 weeks compared to those with no increase in intake [14]. These results should be interpreted with caution, since the confidence interval for one study only narrowly avoided zero. The second meta-analysis concluded, however, that there is only suggestive evidence (standard mean difference: -0.16) to recommend increased intakes for prevention of weight gain [15]. The main outcomes studied for these meta-analyses were different, and therefore the included studies varied. Another systematic review of experimental and longitudinal studies concluded that an inverse relationship between fruit and vegetables intake and adiposity among overweight adults seems to be weak [16].

Various reasons for such equivocal results could include the quality of mass marketed fruit and vegetables produced in large mono-cultural agribusiness, compared with higher nutrient quality produce from mixed low-tech agriculture [17].

RCTs of dietary interventions are limited firstly by not being the correct choice for studies where standardization of one item is impossible. Detailed dietary studies require nutrient metabolomics and wholefood grading appropriate for metabolic health. Mathematical systems modeling can help to deal with the most influential variables. The oft-quoted lack of double blinding, poor compliance, could contain crossover bias [18], high dropout rates as demonstrated by the evaluation of RCT studies in the recent meta-analyses [19, 20]. These are likely signs of inappropriate study design. However, few studies have been undertaken with such food nutrient modeling, so well-designed prospective cohort studies could add important information and provide complementary high level evidence on the research question.

Therefore, the aim of the present systematic review and meta-analysis was to address the relation of habitual fruit and vegetable consumption with subsequent changes in anthropometric variables in adult populations, via analyzing data from prospective cohort studies. Usually, these populations gain weight over time and the outcome of the studies will be gain in anthropometric variables in relation to average gain.

## Materials and Methods

This systematic review was planned and conducted according to the standards of the Meta-analysis of Observational Studies in Epidemiology [21]. Our protocol has been registered in the PROSPERO International Prospective Register of Systematic Reviews (crd.york.ac.uk/prospero/index.asp, identifier: CRD42014013692).

### Literature Search

A systematic search was performed in PubMed (from 1966) and EMBASE (from 1980) for studies published until July 2015. We searched for articles of original research by using the following search terms: ("fruit" OR "vegetable" OR "fruits" OR "vegetables") AND ("weight" OR "waist circumference" OR "obesity" OR "BMI" OR "body mass index" OR "hip" OR "energy intake" OR "energy balance") AND ("longitudinal" OR "prospective" OR "cohort" OR "change" OR "follow-up" OR "nested").

No restrictions to language were made. We manually examined the reference lists from articles eligible for inclusion and from systematic reviews of observational studies [2, 16, 22–24]. The search was conducted independently by three authors (LS, TKU and BB), with disagreements resolved by consensus.

### Eligibility criteria

Studies were included in the meta-analysis if they met all of the following criteria: (i) prospective observational study design with information of at least one measurement of fruit or vegetable intake (starchy tubers were excluded), or both combined, and measurement of change in at least one anthropometric characteristic. Note that only whole fruits were considered, and studies reporting only consumption of fruit juices were excluded); (ii) the primary outcomes were: change in body weight (increase and/or decrease), either measured continuously (g/year) or binary as incident major weight gain (e.g., incidence of gaining a specific amount of weight), and in overweight (Body Mass Index, BMI: ≥25 kg/m^2^) /obesity (BMI: ≥30 kg/m^2^); and the secondary outcomes were: change in waist circumference (increase and/or decrease), and BMI; (iii) reported adjusted beta-coefficients with corresponding standard error, or data necessary to calculate these (95% confidence intervals, (95% CI), standard deviations, p-values); (iv) adjusted odds ratios with corresponding 95% CI or standard error; (v) mean body weight changes with corresponding standard error and (vi) participants were adults (aged 18 years or over) and free of chronic disease (cardiovascular disease, cancer) at study inception. When a study was published in duplicate, we included the version containing the most comprehensive information (latest information in the case of follow-up studies).

### Data extraction and quality assessment

We extracted the following data from each study: first author’s name, publication year, location, cohort name, type of outcome, population (age, sex, ethnicity, and sample size), follow-up duration, dietary assessment method, definition of exposure (including the unit of consumption), whether the exposure was modeled continuously or categorical, outcome definition, outcome assessment, statistical analysis method, and variables adjusted for. Further, we extracted data of the effect sizes with their corresponding uncertainties. When a study reported only sex-specific effect estimates, they were treated as separate studies. When a study provided effect estimates for different degrees of adjustment, those from the most completely adjusted model were chosen. Study quality was assessed in accordance with a recent meta-analysis of sugar intake and adiposity [25], and included risk of bias (selection of exposed and unexposed in cohort studies from different populations; partially flawed measurement of both exposure (i.e. relied on baseline fruit & vegetables consumption alone; measurement error) and outcome (i.e. self-reported); Inconsistency: i.e. point estimates vary widely across studies; confidence intervals shows minimal or no overlap; statistical test for heterogeneity shows a low p-value; I^2^ is large [25].

We followed the guidelines by the GRADE working group, a well-established tool in developing clinical or practice guidelines is assessing the quality and strength of the evidence [26, 27]. We focus especially on risk of bias, and inconsistency of the pooled effects (S2 Table).

### Statistical analysis

Because of differences between studies in the methods used to report on fruit and vegetable consumption and changes in anthropometric measures, we classified the prospective observational studies according to the type of measure of association they used:

1. Beta-coefficients for the association of fruit and vegetable intake at baseline with subsequent changes in anthropometric outcomes;
2. Beta-coefficients for the association of reported changes in fruit and vegetable consumption over time with changes in anthropometric outcomes;
3. Odds ratios for risk of overweight or obesity, or for risk of gaining a particular amount of weight comparing the highest vs. lowest fruit and vegetable intake category;
4. Mean differences of change in anthropometric measures over time comparing the highest and lowest category of fruit and vegetable consumption.

Study-specific results were pooled with random-effects inverse-variance weighted meta-analysis. The weighting of each study was based on the standard error of the corresponding effect size, which was calculated from the 95% confidence intervals (if not provided as such). For outcomes reported on a continuous scale, we re-scaled the reported results to reflect effect sizes associated with each 100-g higher fruit and vegetable intake. When studies reported results according to servings of fruit and vegetables but did not quantify serving size, we assumed a serving of vegetables weights 77 g and that of fruit 80 g, as previously done [28]. When a study reported results for different centers within a country, we pooled those with fixed-effects meta-analysis and meta-analyzed the country-specific estimate (applied for the EPIC study).

In individual meta-analyses we pooled beta-coefficients and mean changes for anthropometric outcomes modeled as continuous variables and odds ratios for outcomes that were binary. This was done for fruit intake, vegetable intake, and for the intake of fruit and vegetables combined. Heterogeneity between study results was evaluated with the Q statistic and the I^2^ statistic. I^2^ values >50% were indicative for substantial heterogeneity across studies [29]. Funnel plots, in which the effect sizes are plotted against their corresponding uncertainty, were used to assess the presence of small study bias if at least 10 studies were available [30]. In addition, an Egger test was performed to test for potential publication bias (“metabias” command) [31]. We used the “metan” command in Stata 12.0 (Stata Corp. 2011, Texas, USA) for all meta-analyses.

## Results

### Literature search and study characteristics

**Fig 1:**
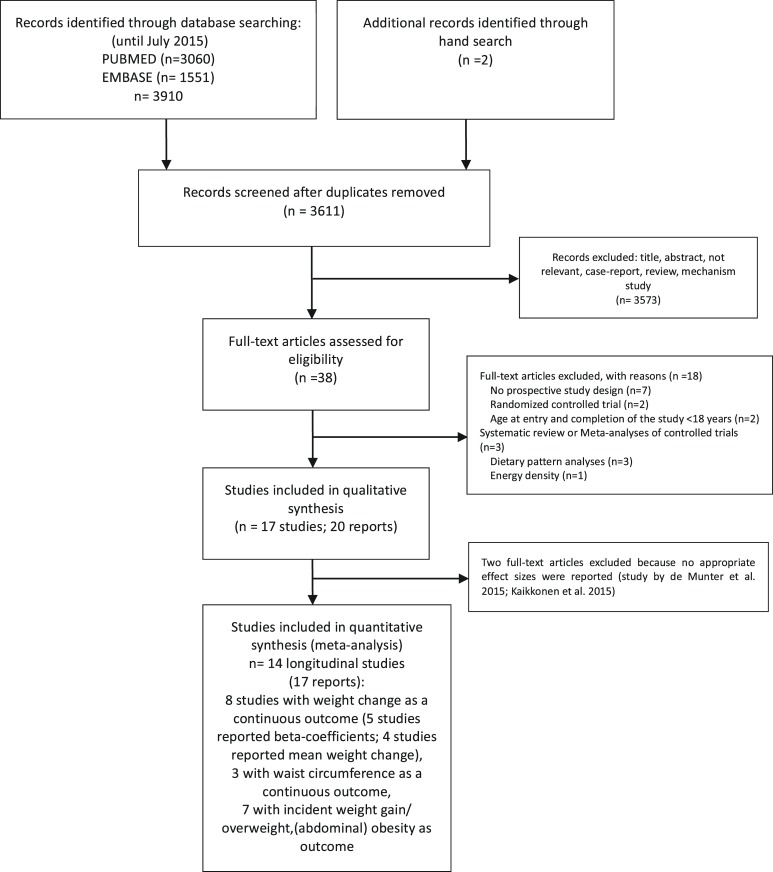
Flow diagram.

Twenty articles reporting on 17 independent studies were included in the systematic review [12, 32–50], and 14 studies provided sufficient data for inclusion in the meta-analysis [12, 32–37, 40–42, 44, 45, 48, 50] (Fig 1 and S1 Table). Eight studies reported on changes in body weight [12, 32, 33, 38, 41, 42, 44, 50], three on changes in waist circumference [33, 35, 40], two on changes in BMI [37, 48], and seven on incident weight gain/ (abdominal) obesity, and overweight [34, 36, 37, 43–45, 50]. Sample size varied between 206 and 233,755 participants, follow-up duration from 9 months to 24 years. Baseline mean BMI at study entry (≥18 years of age) ranged between 22.3 and 27.1 kg/m^2^ (Table 1). The present systematic review included 17 longitudinal studies lasting at least 9 months, in which data relating to an association between either fruit & vegetable indicated in combination (fruit & vegetables) or fruit or vegetables and a measure of adiposity could be extracted.

**Table 1:** Characteristics of prospective observational studies included in the qualitative systematic review or quantitative meta-analysis.

First author	Publication year	Cohort	Country	Sample size	Mean age at entry ± SD (years)	Mean BMI (kg/m2) at entry ± SD (years)	Sex	Diet assessment method	Outcome	Mean Follow-up duration (years)	Adjustment
Aljadani	2013	Australian Longitudinal Study on Women’s Health,	Australia	1356	27.7±1.5	25.2±5.5	W	FFQ (validated)	Changes in body weight by tertiles of F&V intake	6	Physical activity, education, number of births, area of residence, marital status, smoking, and baseline weight, total energy intake
Deforche	2015	n.d,	Belgium	291	17.2±0.5	21.1±2.5 to 22.6±3.1	M/W	FFQ (validated)	changes in BMI per increase of 1 consumption / week of F&V	1.5	n.d
De Munter	2015	Stockholm Public Health Cohort	Sweden	23,108	48.3±16	24.9±3.7	M/W	n.d	Mean changes in BMI: daily (≥1 serving) vs. less than daily (<1 serving) F&V intake	8	Age, education, lifestyle habits, weight status at baseline
Drapeau	2004	Quebec Family Study	Canada	248	39.6±14.2	25.3±4.72	W/M	3-day dietary record	Changes in body weight/ waist circumference according to changes in F over preceding 5 y	5.9	Age, baseline body-weight, or adiposity indicators and changes in daily physical activity level
Esfahani	2014	Teheran Lipid and Glucose Study	Iran	851	M: 40.2 ± 13.5;W: 38.6 ± 11.7	n.d	M/W	FFQ (validated)	Risk of gaining ≥0.5kg weight by servings of F, and V intake	3	Age, body weight, education level, smoking, behaviour, physical activity
Halkjær	2004	MONICA	Denmark	2300	Middle-age	22.8 to 24.7	M/W	FFQ (validated)	Changes in waist circumference by quintiles of F&V/week intake	6	BMI, diet, educational level (three levels), physical activity, smoking status, and alcohol habits
He	2004	NHS I	USA	74,063	49±7 to 52±7	24.8±5 to 25±5	W	FFQ (validated)	Risk of gaining >25kg weight by quintiles of F&V, F, and V intake	12	Age, year of follow-up, physical activity, smoking status, alcohol consumption and caffeine intake, hormone replacement therapy, energy intakes of SFA, PUFA, MUFA, TFA, protein, and total energy and baseline BMI
Holmberg	2013	n.d	Sweden	1322	50.3±7	26.4±3.2	M	FFQ (validated)	Risk of central obesity (WHR: ≥1) for daily vs. less than daily F&V intake	12	None
Kahn	1997	Cancer Prevention Study II	USA	79,236	50–74	M: 25.6±2.6; W: 23.4±3	M/W	FFQ	Changes in BMI and waist by quintiles of V intake; Risk of abdominal obesity by lowest vs. highest V intake	10	Age, education, region of the country, BMI, change in marital status, energy intake, cigarette smoking, meat and vegetable intake, vitamin E use, alcohol intake, physical activity, for women, menopausal status, estrogen use, and parity.
Kaikkonen	2015	Young Finns Study	Finland	1715	24–39	M: 25.64±3.9; W: 24.38±4.5	M/W	FFQ	Changes in weight, and BMI (baseline) by monthly use of F, and V intake (portions)	6	n.d
Mozaffarian	2011	NHS I,	USA	50,422	52.2±7.2	23.7±1.4	W	FFQ (validated)	Changes in body weight; by serving number increase in F, and V intake	20	Age, BMI, television watching, sleep duration, physical activity, alcohol use, smoking, and all of the dietary factors
Mozaffarian,	2011	NHS II,	USA	47,898	37.5±4.1	23±2.7	W	FFQ (validated)	Changes in body weight by serving number increase in F, and V intake	12	Age, BMI, television watching, sleep duration, physical activity, alcohol use, smoking, and all of the dietary factors
Mozaffarian,	2011	HPS	USA	22,557	50.8±7.5	24.7±1.1	M	FFQ (validated)	Changes in body weight by serving number increase in F, and V intake	20	Age, BMI, television watching, sleep duration, physical activity, alcohol use, smoking, and all of the dietary factors
Nikolaou	2014	n.d	United Kingdom	1275	20±3.6	22.3±4.6	W/M	FFQ (validated)	Changes in body weight for meeting ‘5-a-day’-F&V goal versus not meeting it	<1	Baseline weight, height, age, and gender
Rautiainen	2015	WHS	USA	18,146	52±6.2 to 55±7.7	22.4±1.6	W	FFQ (validated)	Risk of overweight and obesity quintiles of F&V, F, and V intake. Changes in body weight by quintiles of F&V, F, and V (servings) intake	17	Age, randomization treatment assignment, physical activity, history of hypercholesterolemia and hypertension, smoking status, postmenopausal status, hormone use, multivitamin use, and energy intake
Romaguera;Nooyens;Halkjæ	2011; 2005; 2009	EPIC	5 European countries	48,631	Exclusion baseline >60 years; and follow-up >65	n.d	M/W	FFQ (validated)	Changes in waist circumference per 100 kcal higher F, and V intake	5.5	Total energy intake, age, baseline weight, baseline height, baseline WCBMI, smoking, alcohol intake (except in the models including alcoholic beverages), physical activity, education, follow-up duration, menopausal status (women only), and hormone replacement therapy use (women only)
Sanchez-Villegas	2006	SUN	Spain	6319	34±10.1 to 40±13.1	23.4±3.4	M/W	FFQ (validated)	Changes in body weight per tertile F&V intake	2	Age, gender, baseline BMI, smoking, physical activity, alcohol consumption, energy intake, change in dietary habits
Vergnaud; Buijsse	2012; 2009	EPIC	10 European countries	233,755	25–70	24.8±4.2 to 27.1 ±3.6	M/W	FFQ (validated)	Changes in body weight per 100-g higher F&V, F, and V intake; Risk of weight gain (≥ 1 kg) per 100 g increase in F&V intake	5	Age and an indicator of vegetable (or fruit) consumption, educational level, physical activity level, smoking status, BMI, follow-up time, energy intake, energy intake from alcohol, plausibility of total energy intake reporting, and fruit (for vegetable analysis) or vegetable (for fruit analysis) intake
Vioque	2008	n.d,	Spain	206	41.52 ±17.9	25.82±4.6	M/W	FFQ (validated)	Risk of gaining ≥3.41 kg by quartiles of F and V intake	10	Sex, age, educational level, BMI, time spend watching TV, presence of disease, height, total energy, and energy-adjusted intakes of protein, SFA, MUFA, PUFA, fiber, caffeine, and alcohol consumption; self-reported change of fruit intake over the past 10 years and the self-reported change of vegetable intake over past 10 years

BMI: body mass index; F: fruits; F&V: fruits & vegetables; FFQ: food frequency questionnaire; MUFA: monounsaturated fatty acids; n.d., no data; PUFA: polyunsaturated fatty acids; SFA: saturated fatty acids; TFA: total fatty acids; V: vegetables; WHR: waist-to-hip ratio

The total number of participants in the present systematic review was 563,277. All but two studies were conducted in the North America and Europe. Fruit & vegetable, fruit, or vegetable consumption was reported in grams, servings, percentage of energy intake, frequency of consumption, or in quantile categories. Measures of association were coefficients from linear regression analysis, mean changes or odds ratios. All but three studies used food-frequency questionnaires to estimate dietary intake.

Of these 17 studies, eight used self-reported estimates of body weight or waist circumference during base line and follow up [12, 32, 35, 36, 38, 41, 47, 50], and the EPIC study a mix between the types of information generation [40, 42]; three studies collected exposure data from questionnaires without considering the validity for assessing fruit & vegetable consumption in the analytical model [33, 37, 47]; and 8 out of 17 studies provided estimates that were adjusted for total energy intake [32, 36, 37, 40–42, 44, 50]. There was inconsistency in the covariates used to adjust analyses and a wide range of methods of assessing fruit & vegetable intake and adiposity outcomes, which made pooling of studies difficult. Four studies reported a significant loss of body weight with increasing fruit and/or vegetable intake ranging from 0.001 to 1.6 kg [12, 32, 33, 42], while one study reported an average gain in body weight of 0.21 kg but this result was not significant [38]. Two studies reported a loss in waist circumference ranging from 0.01 to 0.19 cm with increased intake of fruit & vegetable by 100 g/d [33, 40]. One study reported a decrease in BMI by 0.12 kg/ht^2^ for the highest quintile of vegetable intake [37], and another study observed a higher BMI among those with less daily fruit intake compared with lower levels of daily fruits intake [49]. A further four studies reported a significant lower OR for adiposity risk with a higher fruit & vegetable intake with OR values ranging from 0.26 to 0.91 [36, 37, 44, 50].

Consumption of fruit and vegetables combined was inversely associated with changes in body weight in four studies [32, 36, 42, 44]. The EPIC study reported a significant association in men only [42]. Fruit consumption was inversely associated in three studies (EPIC study: significant association only for women) [12, 33, 42], and vegetable consumption was inversely associated in three studies (EPIC: significant association only in men) [12, 36, 42].

### Changes in body weight

**Fig 2:**
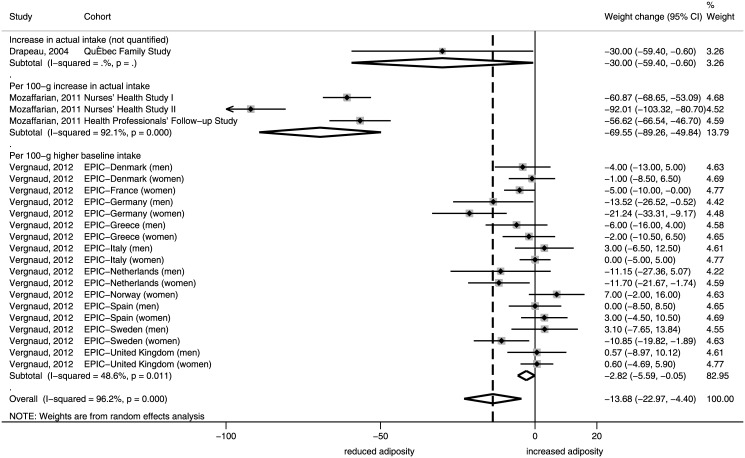
Forest plot of associations between changes in body weight (g/year) and fruit consumption in cohort studies of adults. I^2^: Inconsistency.

For fruit, we pooled effect sizes of 4 North-American studies and of 18 sex- and country-specific estimates from the EPIC study. After pooling these studies, each 100-g higher F intake was inversely associated with weight change (decrease), with the combined regression coefficient of -13.68 g/year (95% CI, -22.97 to -4.40). There was large heterogeneity between studies (I^2^ = 96.2%), which was mostly due to differences between the North American and European effect sizes (Fig 2).

**Fig 3:**
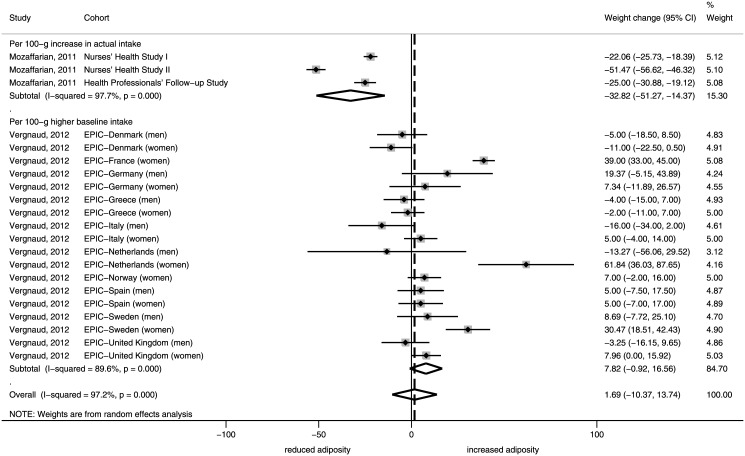
Forest plot of associations between changes in body weight (g/year) and vegetable consumption in cohort studies of adults. I^2^: Inconsistency.

4 studies with 494,680 participants considered the association between vegetable consumption and weight change. Every 100-g increase in vegetables was associated with a non-significant 1.69 g/year (95% CI, -10.37 to 13.74; I^2^ = 97.2%) change (increase) in body weight (Fig 3).

Since the exposure was defined differently on fruit & vegetable intake and body weight changes for three studies, we presented the results only via systematic review (no meta-analysis).

The Australian Longitudinal Study on Women’s Health detected an inverse association comparing the highest vs. lowest intake fruit & vegetable intake category (-266 g/y). A study among first-year students showed no significant association between weight meeting “5-a-day” vs. less than “5-a-day” of fruit and vegetables and changes in body weight. Moreover the EPIC study indicated that higher baseline fruit & vegetable intake was not associated with changes in body weight (significant inverse association only for men).

### Changes in waist circumference and BMI

There was significant inverse association between higher intake of whole fruit and changes (decrease) in waist circumference (beta: -0.03 cm/year, 95% CI, -0.06 to -0.00) (S1 Fig). Furthermore, a significant association between a 100 kcal increase in fruit intake and changes (decrease) in waist circumference (beta: -0.04 cm/year, 95% CI, -0.05 to -0.03; I^2^ = 60.6%) could be observed, respectively. Pooling male and female participants from one cohort study showed that the highest quintile of vegetable intake was associated with a significant lower increase in BMI (beta: -0.01 units/year, 95% CI, -0.02 to -0.01; I^2^ = 0%). No significant association was observed for combined fruit & vegetable consumption and change in waist circumference.

### Risk of weight gain or abdominal obesity

Comparing the highest fruit & vegetable, fruit, and vegetable intake categories were associated with a 9%, 17%, and 17% reduced risk of adiposity (OR: 0.84, 95% CI, 0.91 to 0.99; I^2^ = 53%), (OR: 0.83, 95% CI, 0.71 to 0.99; I^2^ = 74.6%), and (OR: 0.83, 95% CI, 0.70 to 0.99; I^2^ = 28%), respectively (S2–S4 Figs).

### Mean weight change in body weight

Pooling four cohort studies showed no significant differences between the highest vs. lowest intakes of fruit & vegetable / fruit/ vegetable on changes in body weight (mean difference: -0.13 kg, 95% CI -0.40 to 0.15, I^2^ = 77.1%).

### Study quality, methodological issues, and sensitivity analyses

Since we observed significant heterogeneity for the 100g increase in fruit or vegetable on comparisons, we performed sensitivity analyses excluding the Nurses’ Health Study I+II, and the Health Professionals’ Follow-up Study as these studies provided no quantitative information about a serving size. For fruit, heterogeneity could be significantly reduced (I^2^ = 43.2%). However, heterogeneity for a 100g-increase in vegetables intake remained high. We thus compared the vegetable intakes in EPIC for southern (Italy, Greece, Spain) vs. other countries. Including only the southern countries resulted in low heterogeneity (I^2^ = 18.9%).

Overall, studies that showed an inverse association between fruit & vegetable followed participants over longer periods of time [12, 32, 33, 36, 37, 40, 44, 50], than the ones showing no association [34, 38, 41].

Inspection of the funnel plots (only the subgroups for 100g increase in fruit or vegetable provided at least 10 studies) showed low to moderate asymmetry; however, in view of the between-study heterogeneity, this result need careful interpretation.

## Discussion

The results of the present meta-analysis showed that a higher fruit intake was associated with a smaller weight gain. Overall this suggested a 13g to 14g smaller weight gain per year for a daily higher intake of 100g fruit. The benefit seems to be low, considering a difference of ≈300g (fruit) between the highest vs. lowest tertiles of fruit intake. Low fruit intakes would roughly account for approximately 6% of the overall weight change of 450g/y observed in those with low intakes. Taking the odds ratio into account, the meta-analysis showed that fruit & vegetable, fruits, and vegetable intake had additional beneficial effects on adiposity outcomes (reduced risk: 9–17%). In addition higher intakes of fruit and vegetable were inversely related to changes in waist circumference.

Prospective cohort studies have shown that weight change is an important outcome marker, and population metric of increased or decreased energy balance [12]. However, measures including waist such as waist/hip AND/OR waist/height are much better indexes for metabolic health [51, 52]. Overall, fruit & vegetable consumption either induces a decrease in body weight or a lower weight gain as part of a larger dietary change pattern that includes intakes of less energy dense foods, and higher intakes of fibre and associated micronutrients. All these associations must be mediated by changes in energy intake, energy expenditure (or both), or change in fraction of energy that is absorbed from food. Data from intervention trials showed that overall dietary changes (lower consumption of processed food, refined carbohydrate) were associated higher consumption of fruit and vegetables [53, 54].

Recent studies are showing that food that is both naturally micronutrient dense, and moderately energy dense, such as found in traditional Mediterranean diets, reduces waist circumference, metabolic disease risk and to a lesser degree weight. As peripheral adipose mass, which may be large and the most significant contributor to weight in some ethnicities, is metabolically benign, the metabolically stabilizing micronutrients in fruit and vegetables may not show weight loss in those with this genetic adipose distribution. However, mostly importantly, increased fruit and vegetable intake is shown to reduce weight gain in all body types. This fact may help explain the several studies that have shown that when fruit & vegetable consumption increased without a change in energy intake, weight loss did not occur [55, 56]. Another possible biological explanation of the lower weight gain could be also the fiber content of fruit & vegetable. Fiber, which is also a major carrier of micronutrients, is known to slow digestion and augment satiety [57]. A recent report from the EPIC study showed however, that fiber from fruit and vegetables was not associated with body weight changes, but was with significant changes in waist circumference [58].

Therefore it is not surprising that a recent meta-analysis showed a non-significant effect on energy intake for an intervention with high fruit & vegetable intake compared to the control group, but a significant weight change [14]. Another meta-analysis detected no significant reduction in body weight including randomized controlled trials promoting higher intakes of fruit & vegetables (this meta-analysis with very real possibility of funding bias) [15]. With all of the above studies, nutrient intake, including energy, is notoriously poorly estimated from all methods of data collection (FFQ) by self-conscience and sensitized study participants in free-living conditions, so meta-analyses reflect this. One study showed that the comparability and reliability of FFQs over a time period of three months was highly reproducible [59]. Moreover, the correlation coefficient for fruits was 0.63 [60] and for vegetables ranged between 0.74 to 0.81 in adult populations [61, 62].

A report by the WHO/FAO expert consultation activity on diet, nutrition and prevention of chronic disease from 2003, sets population nutrient goals and recommends an intake of a minimum of 400g of fruits & vegetables per day to prevent chronic diseases including obesity [63]. The report stated that there is convincing evidence that fruits and vegetables reduce the risk of obesity [64]. The Dietary guidelines for Americans 2010 stated that there is moderate evidence in adults that increased intakes of vegetables and/or fruits may protect against weight gain [1]. Although these statements might have been valid at the time of their publication, they could not be fully confirmed throughout our systematic review. Strong statements should be clearly reflected by consistent and substantial effects in study results, which was not the case following analysis of the prospective studies available for this meta-analysis. However, none of the study analyses were particularly planned at the beginning of the cohort study. In nearly all cases addressing weight change as outcome was considered during the course of the study and probably came about due to reassessment or re-measurement of anthropometry. According to the GRADE guidelines, the overall quality of the obtained evidence needs to be considered as low [26]. Another systematic review on nutrients and foods and long-term weight change with no particular focus on fruit & vegetable concluded that there was suggestive evidence for a link between fruit intake and protection against larger increases in waist circumference [65]. This statement is more in line with our results than the previous conclusions.

The world’s largest lifestyle intervention trial, the Women’s Health Initiative Dietary Modification Trial including 48 835 postmenopausal women not only focused on increased intakes of fruit and vegetables, but also on reduced intakes of fat, however observing no significant weight reduction compared to the control group over a period of 7.5 years of follow up. This could be due to the high carbohydrate content of low fat diets [66]. Long-term intervention trials focusing only on fruits and/or vegetables intakes are rare. A recent 12-month single blind parallel controlled trial including 120 overweight adults investigated the effects of two energy-reduced diets differing only by doubling the serving size of vegetables. A greater weight reduction in the higher vegetable group could only be observed in the short term (3 months). At 12 months both groups reduced their body weight by approximately 6.5 kg [67]. Similar results could be observed in another 12-month intervention trial on increased fruit and vegetables consumption [68]. Another recent review of the evidence including 16 randomized controlled trials concluded that a higher vegetable consumption in a healthy diet may prove beneficial for weight loss in overweight adults [67].

Even with such poor characterization of diet such as the inclusion of low nutrient/high starch vegetables such as potato in some studies, and of body fat distribution (weight includes those with healthy peripheral and unhealthy central fat) that change to, or higher habitual fruit & vegetable diets almost always prevents weight gain, or decreases waist. In addition, other metabolic improvements (blood pressure, blood glucose and lipids) all align with evolutionary evidence of healthy human nutrition, as noted above. The findings of this systematic review and meta-analysis support increasing fruit & vegetable as a public health measure to prevent cardiovascular disease and cancer [2, 7].

### Limitations

The present systematic review has several limitations due to the quality of the data of the studies and heterogeneity between studies. First, the assessment of body weight at baseline and follow-up differed across cohorts (e.g. at follow-up in some cohorts participants were weighed, in other cohorts participants self-reported their body weight). The Harvard cohorts compared people who increased their fruit & vegetable intake over time versus those who did not, which is a better design than comparing differences in intake between people assessed a single time at baseline. Furthermore it seems that methodological limitations are more prevalent in these studies, and sample size seems to be lower, however some discrepancies remain [42].

Several studies relied only on baseline fruit & vegetable consumption and therefore assume a stable consumption over time. Furthermore, measurement errors on dietary intake are common in prospective cohort studies, with validity and reliability being lower compared to randomized controlled trials, since dietary intake is usually calculated from self-assessment tools such as FFQs [69]. The low number of studies that finally could be included in the quantitative meta-analysis is another clear limitation. Furthermore, the included studies were heterogeneous with respect to the population size, follow-up length, baseline age, fruit & vegetable categories, fruit & vegetable definition (some included potatoes or nuts), adjustment factors, and outcome estimates, and the meta-analysis showed significant heterogeneity. Socio-demographic or lifestyle factors, such as education and physical activity are associated with lower body weight/adiposity and higher fruit and vegetable intake [70–72]. Twelve cohort studies were adjusted for physical activity, and nine studies for educational level. The Harvard cohorts included health professionals, the SUN cohort university graduates, and the study by Nikolaou et al. first year university students. These heterogeneous estimates could also be detected in recent meta-analyses of intervention trials [14, 15]. In addition, there might have been some kind of “geographical bias”, i.e. most of the studies were conducted in North America and Europe and cannot be generalized to other parts of the world, including southern countries where the consumption of fruit and vegetables is high.

## Conclusion

We could show that taking all empirical study data into account, fruit is related to body weight changes (-13.68 g/y), and fruit & vegetable, fruit and vegetable intake inversely associated (9–17% reduced risk) with risk of overweight, (abdominal) obesity/weight gain. Although the overall quality of the evidence is considered to be low following their assessment via the GRADE guidelines, the present findings might have public health relevance and support initiatives to increase the intake of fruit and vegetables. More high quality research from randomized controlled trials, and well-designed prospective cohort studies are needed to clarify the effects of fruit and vegetables on changes in anthropometric outcomes.

## Supporting Information

S1 Fig Forest plot of associations between changes in waist circumference (cm/year) and fruit and vegetable consumption in cohort studies of adults. (DOCX)

S2 Fig Forest plot showing pooled odds ratio (OR) with 95% CI for weight gain/ overweight, (abdominal) obesity comparing categories of vegetable intakes. (DOCX)

S3 Fig Forest plot showing pooled odds ratio (OR) with 95% CI for weight gain/ overweight, (abdominal) obesity comparing categories of fruit and vegetable consumption. (DOCX)

S4 Fig Forest plot showing pooled odds ratio (OR) with 95% CI for weight gain/ overweight, (abdominal) obesity comparing categories of fruit intakes. (DOCX)

S1 Table Full-text articles excluded with reasons. (DOCX)

S2 Table GRADE summary of findings table for the effect of an increase in F&V, F or V intake in adults (Quality assessment was performed if at least 3 studies/outcome were available). (DOCX)
