# Supplementary material for: Fruit and Vegetable Consumption and Changes in Anthropometric Variables in Adult Populations: A Systematic Review and Meta-Analysis of Prospective Cohort Studies
Source: PLoS One. 2015 Oct 16;10(10):e0140846. doi: 10.1371/journal.pone.0140846 (PMC4608571; doi:10.1371/journal.pone.0140846)
Supplement: S1 Table — (DOCX) [file pone.0140846.s005.docx]

S1 Table: Full-text articles excluded with reasons:

| **Reference** | **Reason for exclusion** |
| --- | --- |
| Linde JA, Utter J, Jeffery RW, Sherwood NE, Pronk NP, Boyle RG. Specific food intake, fat and fiber intake, and behavioral correlates of BMI among overweight and obese members of a managed care organization. *International Journal of Behavioral Nutrition and Physical Activity* 2006; 3: 42. | Randomized controlled trial |
| [Stamler J](http://www.ncbi.nlm.nih.gov/pubmed/?term=Stamler%20J%5BAuthor%5D&cauthor=true&cauthor_uid=8988948), [Dolecek TA](http://www.ncbi.nlm.nih.gov/pubmed/?term=Dolecek%20TA%5BAuthor%5D&cauthor=true&cauthor_uid=8988948). Relation of food and nutrient intakes to body mass in the special intervention and usual care groups in the Multiple Risk Factor Intervention Trial. Am J Clin Nutr. 1997 Jan;65(1 Suppl):366S-373S. | Randomized controlled trial |
| Chan R, Chan D, and Woo J. Associations between dietary patterns and demographics, lifestyle, anthropometry and blood pressure in Chinese community-dwelling older men and women. *Journal of Nutritional Science* 2012; 1: e20. | Cross-sectional study |
| Davis JN, Hodges VA, Gillham MB. Normal-weight adults consume more fiber and fruit than their age- and height-matched overweight/obese counterparts. J Am Diet Assoc 2006; 106: 833–  840 | Cross-sectional study |
| Lin BH, Morrison RM. Higher fruit consumption linked with lower body mass index. *Food Review* 2002; **25**: 28–32. | Cross-sectional study |
| Moreira P, Padrao P. Educational, economic and dietary determinants of obesity in Portuguese adults: a cross-sectional study. *Eat Behav* 2006; **7**: 220–228 | Cross-sectional study |
| Trudeau E, Kristal AR, Li S, Patterson RE. Demographic and psychosocial predictors of fruit and vegetable intakes differ: implications for dietary interventions. *J Am Diet Assoc* 1998; **98**: 1412–  1417. | Cross-sectional study |
| Serdula MK, Byers T, Mokdad AH, Simoes E, Mendlein JM, Coates RJ. The association between fruit and vegetable intake and chronic disease risk factors. *Epidemiology* 1996; **7**: 161–165. | Cross-sectional study |
| Kasparek DG, Corwin SJ, Valois RF, Sargent RG, Morris RL. Selected health behaviors that influence college freshman weight change. *J Am Coll Health* 2008; 56: 437–444. | years of age <18 years |
| te Velde SJ, Twisk JW, Brug J (2007) Tracking of fruit and vegetable consumption from adolescence into adulthood and its longitudinal association with overweight. Br J Nutr 98: 431-438. | years of age <18 years |
| Ledoux TA, Hingle MD, Baranowski T. Relationship of fruit and vegetable intake with adiposity: a systematic review Obesity Reviews 2011, 12: e143–e150. doi: 10.1111/j.1467-789X.2010.00786.x | Systematic review |
| Kaiser KA., Brown AW, Brown MMB, Shikany JM., Mattes RD, Allison DB. Increased fruit and vegetable intake has no discernible effect on weight loss: a systematic review and meta-analysis. *Am J Clin Nutr* 2014, 100(2): 567-576. | Systematic review and meta-analysis |
| Mytton OT, Nnoaham K, Eyles H, Scarborough P, Mhurchu CN. Systematic review and meta-analysis of the effect of increased vegetable and fruit consumption on body weight and energy intake. *BMC public health* 2014; 14(1): 886. | Systematic review and meta-analysis |
| Quatromani PA, Copenhafer DL, D’agostino RB, Millen BE. Dietary patterns predict the development of overweight in women: The Framingham Nutrition Studies. *Journal of the American Dietetic Association* 2002, 102: 1239-1246. | Cluster analysis |
| Newby PK, Muller D, Hallfrisch J, Qiao N, Andres R, Tucker KL. Dietary patterns and changes in body mass index and waist circumference in adults. *The American journal of clinical nutrition* 2003, 77: 1417-1425. | Cluster analysis |
| Togo P, Osler M, Sorensen TI, Heitmann BL. A longitudinal study of food intake patterns and obesity in adult Danish men and women. *Int J Obes Relat Metab Disord* 2004 28:583–593 | Factor analysis |
| Savage JS, Marini M, Birch LL. Dietary energy density predicts women's weight change over 6-y. *The American journal of clinical nutrition* 2008, 88: 677-684. | Energy density |
| Parker DR, Gonzalez S, Derby CA, Gans KM, Lasater TM, Carleton RA. Dietary factors  in relation to weight change among men and women from two south-eastern New England communities. *Int J Obes Relat Metab Disord* 21:103–109 | No prospective cohort design |
