# Supplementary material for: Fruit and Vegetable Consumption and Changes in Anthropometric Variables in Adult Populations: A Systematic Review and Meta-Analysis of Prospective Cohort Studies
Source: PLoS One. 2015 Oct 16;10(10):e0140846. doi: 10.1371/journal.pone.0140846 (PMC4608571; doi:10.1371/journal.pone.0140846)
Supplement: S2 Table — (DOCX) [file pone.0140846.s006.docx]

| Quality Assessment | | | | | No of participants | Effect | Quality |
| --- | --- | --- | --- | --- | --- | --- | --- |
| No of studies | Design | Risk of bias | Inconsistency | Other considerations | overall | Absolute (95% CI) |  |
| Q1: What is the effect of an increase in F on beta coefficients for changes in body weight in adults? | | | | | | | |
| 5 | Cohort | serious | serious | None | 354,880 | beta: -13,68 g/y (-22,97 to -4,40) | Low |
| Q2: What is the effect of an increase in V on beta coefficients for changes in body weight in adults? | | | | | | | |
| 4 | Cohort | serious | very serious | None | 354,632 | beta: 1,69 g/y (-10,37 to 13,47) | Low |
| Q3: What is the effect of an increase in F&V on OR for weight gain or risk of (abdominal) obesity in adults? | | | | | | | |
| 5 | Cohort | serious | serious | None | 183,169 | Odds Ratio: 0,91 (0,84 to 0,99) | Low |
| Q4: What is the effect of an increase in F on OR for weight gain or risk of (abdominal) obesity, overweight in adults? | | | | | | | |
| 4 | Cohort | serious | not serious | None | 93,266 | Odds Ratio: 0,83 (0,71 to 0,99) | Low |
| Q5: What is the effect of an increase in V on OR for weight gain or risk of (abdominal) obesity, overweight in adults? | | | | | | | |
| 5 | Cohort | serious | serious | None | 172,502 | Odds Ratio: 0,83 (0,70 to 0,99) | Low |

S2 Table: GRADE summary of findings table for the effect of an increase in F&V, F or V intake in adults (Quality assessment was performed if at least 3 studies/outcome were available)

Risk of bias: i.e. selection of exposed and unexposed in cohort studies from different populations; partially flawed measurement of both exposure (i.e. relied on baseline F&V consumption alone; measurement error) and outcome (i.e. self-reported)

Inconsistency: i.e. point estimates vary widely across studies; confidence intervals shows minimal or no overlaps; statistical test for heterogeneity shows a low p-value; I^2^ is large

The fact that any potential bias, inconsistency, indirectness, imprecision or reliance on study type other than randomised trials results in downgrading of the quality of evidence means formally identifying effects which are regarded as important and based on high quality evidence using the GRADE system may be unattainable in the context of nutritional determinants of disease. This needs to be taken into account when developing nutritional recommendations.
