# Supplementary material for: Fruit and Vegetable Consumption and Changes in Anthropometric Variables in Adult Populations: A Systematic Review and Meta-Analysis of Prospective Cohort Studies
Source: PLoS One. 2015 Oct 16;10(10):e0140846. doi: 10.1371/journal.pone.0140846 (PMC4608571; doi:10.1371/journal.pone.0140846)
Supplement: Supplementary file 3 [file pone.0140846.s001.docx]

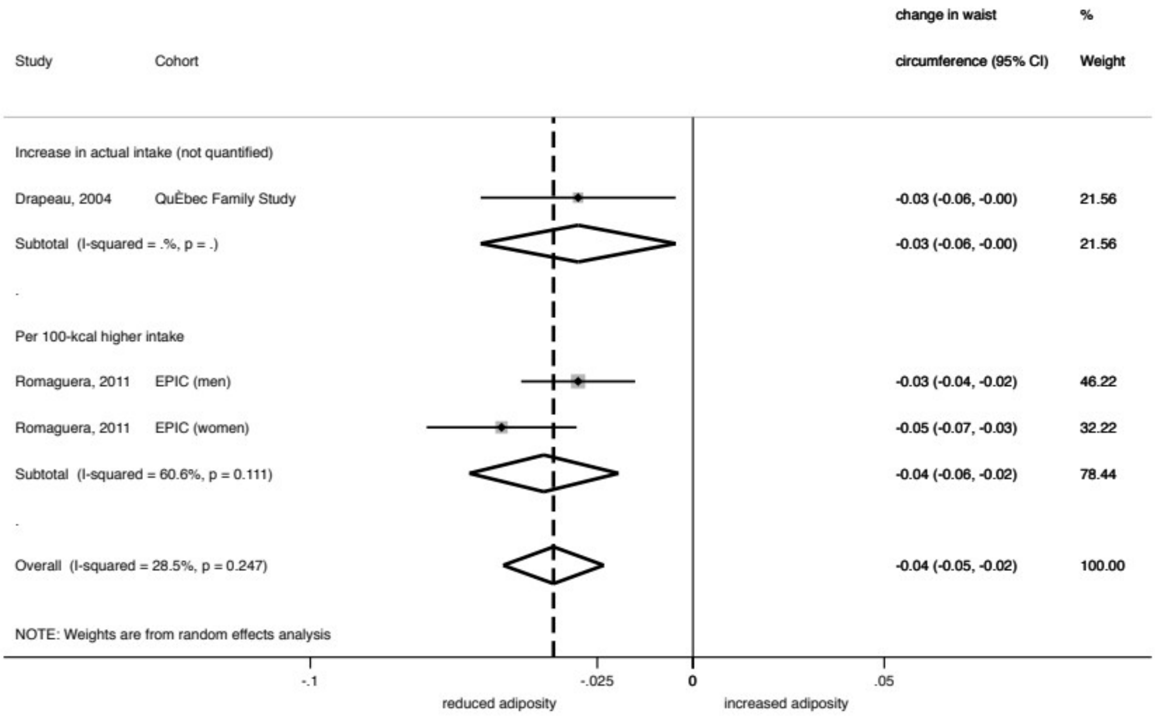
change in waist
%
Study
Cohort
circumference (95% CI)
Weight
Increase in actual intake (not quantified)
Drapeau, 2004
QuÈbec Family Study
-0.03 (-0.06, -0.00)
21.56
Subtotal (I-squared = .%, p = .)
-0.03 (-0.06, -0.00)
21.56
.
Per 100-kcal higher intake
Romaguera, 2011
EPIC (men)
-0.03 (-0.04, -0.02)
46.22
Romaguera, 2011
EPIC (women)
-0.05 (-0.07, -0.03)
32.22
Subtotal (I-squared = 60.6%, p = 0.111)
-0.04 (-0.06, -0.02)
78.44
.
Overall (I-squared = 28.5%, p = 0.247)
-0.04 (-0.05, -0.02)
100.00
NOTE: Weights are from random effects analysis
-.1
-.025
0
.05
reduced adiposity
increased adiposity
