# Supplementary material for: Fruit and Vegetable Consumption and Changes in Anthropometric Variables in Adult Populations: A Systematic Review and Meta-Analysis of Prospective Cohort Studies
Source: PLoS One. 2015 Oct 16;10(10):e0140846. doi: 10.1371/journal.pone.0140846 (PMC4608571; doi:10.1371/journal.pone.0140846)
Supplement: Supplementary file 4 [file pone.0140846.s002.docx]

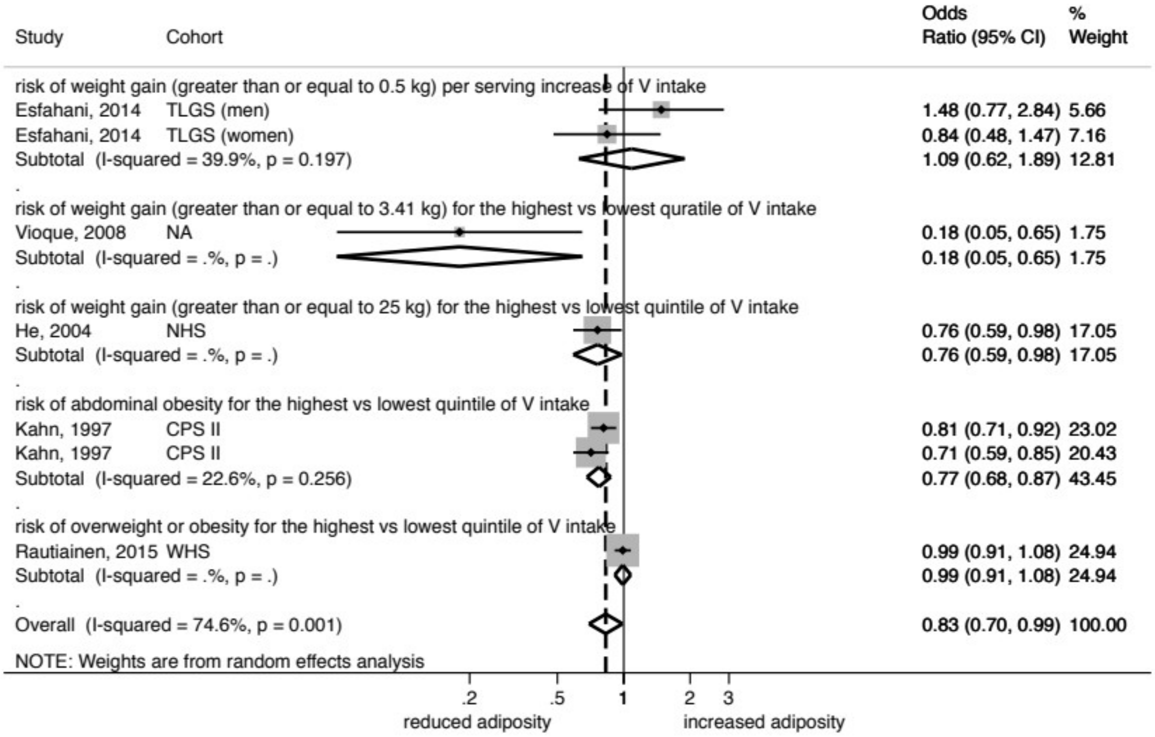

Study
Cohort
Odds Ratio (95% CI)
% Weight
risk of weight gain (greater than or equal to 0.5 kg) per serving increase of V intake
Esfahani, 2014 TLGS (men) 1.48 (0.77, 2.84) 5.66
Esfahani, 2014 TLGS (women) 0.84 (0.48, 1.47) 7.16
Subtotal (I-squared = 39.9%, p = 0.197) 1.09 (0.62, 1.89) 12.81
risk of weight gain (greater than or equal to 3.41 kg) for the highest vs lowest quratile of V intake
Vioque, 2008 NA 0.18 (0.05, 0.65) 1.75
Subtotal (I-squared = .%, p = .) 0.18 (0.05, 0.65) 1.75
risk of weight gain (greater than or equal to 25 kg) for the highest vs lowest quintile of V intake
He, 2004 NHS 0.76 (0.59, 0.98) 17.05
Subtotal (I-squared = .%, p = .) 0.76 (0.59, 0.98) 17.05
risk of abdominal obesity for the highest vs lowest quintile of V intake
Kahn, 1997 CPS II 0.81 (0.71, 0.92) 23.02
Kahn, 1997 CPS II 0.71 (0.59, 0.85) 20.43
Subtotal (I-squared = 22.6%, p = 0.256) 0.77 (0.68, 0.87) 43.45
risk of overweight or obesity for the highest vs lowest quintile of V intake
Rautiainen, 2015 WHS 0.99 (0.91, 1.08) 24.94
Subtotal (I-squared = .%, p = .) 0.99 (0.91, 1.08) 24.94
Overall (I-squared = 74.6%, p = 0.001) 0.83 (0.70, 0.99) 100.00
NOTE: Weights are from random effects analysis
.2
.5
1
2
3
reduced adiposity
increased adiposity
