# Supplementary material for: Fruit and Vegetable Consumption and Changes in Anthropometric Variables in Adult Populations: A Systematic Review and Meta-Analysis of Prospective Cohort Studies
Source: PLoS One. 2015 Oct 16;10(10):e0140846. doi: 10.1371/journal.pone.0140846 (PMC4608571; doi:10.1371/journal.pone.0140846)
Supplement: Supplementary file 5 [file pone.0140846.s003.docx]

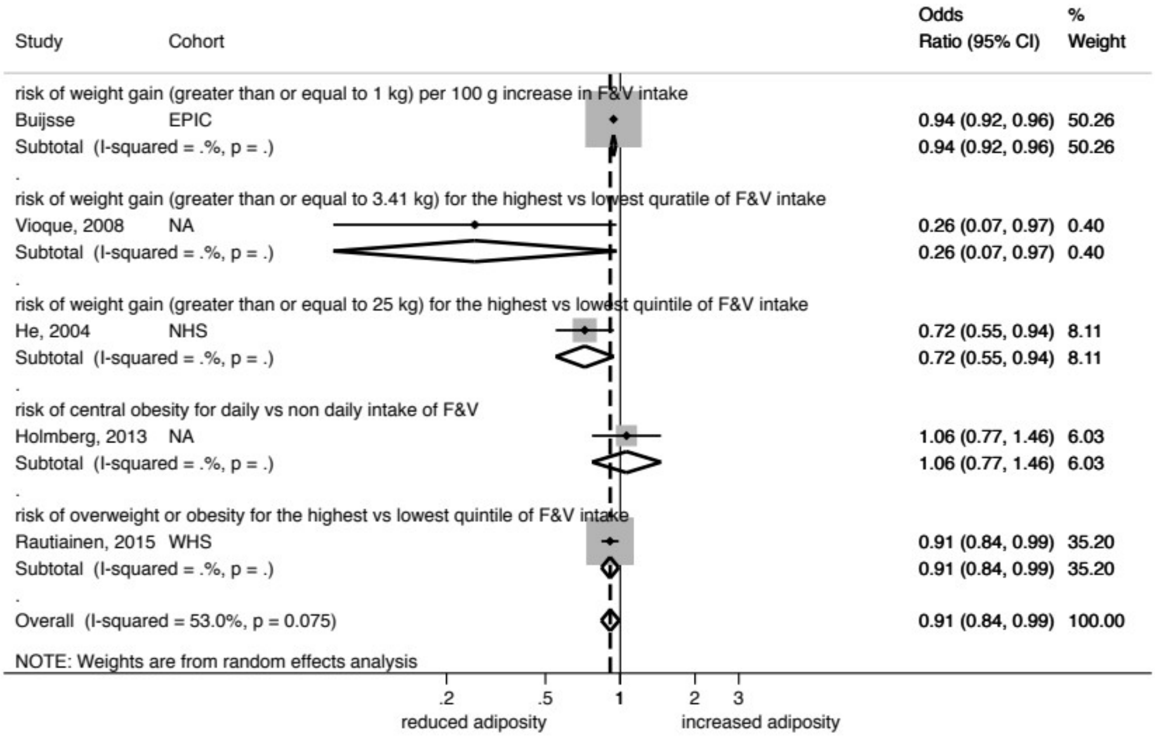
Study
Cohort
Odds Ratio (95% CI)
% Weight
risk of weight gain (greater than or equal to 1 kg) per 100 g increase in F&V intake
Buijsse EPIC 0.94 (0.92, 0.96) 50.26
Subtotal (I-squared = .%, p = .) 0.94 (0.92, 0.96) 50.26
.
risk of weight gain (greater than or equal to 3.41 kg) for the highest vs lowest quratile of F&V intake
Vioque, 2008 NA 0.26 (0.07, 0.97) 0.40
Subtotal (I-squared = .%, p = .) 0.26 (0.07, 0.97) 0.40
.
risk of weight gain (greater than or equal to 25 kg) for the highest vs lowest quintile of F&V intake
He, 2004 NHS 0.72 (0.55, 0.94) 8.11
Subtotal (I-squared = .%, p = .) 0.72 (0.55, 0.94) 8.11
.
risk of central obesity for daily vs non daily intake of F&V
Holmberg, 2013 NA 1.06 (0.77, 1.46) 6.03
Subtotal (I-squared = .%, p = .) 1.06 (0.77, 1.46) 6.03
.
risk of overweight or obesity for the highest vs lowest quintile of F&V intake
Rautiainen, 2015 WHS 0.91 (0.84, 0.99) 35.20
Subtotal (I-squared = .%, p = .) 0.91 (0.84, 0.99) 35.20
.
Overall (I-squared = 53.0%, p = 0.075) 0.91 (0.84, 0.99) 100.00
NOTE: Weights are from random effects analysis
.2
.5
1
2
3
reduced adiposity
increased adiposity
