# Supplementary material for: Fruit and Vegetable Consumption and Changes in Anthropometric Variables in Adult Populations: A Systematic Review and Meta-Analysis of Prospective Cohort Studies
Source: PLoS One. 2015 Oct 16;10(10):e0140846. doi: 10.1371/journal.pone.0140846 (PMC4608571; doi:10.1371/journal.pone.0140846)
Supplement: Supplementary file 6 [file pone.0140846.s004.docx]

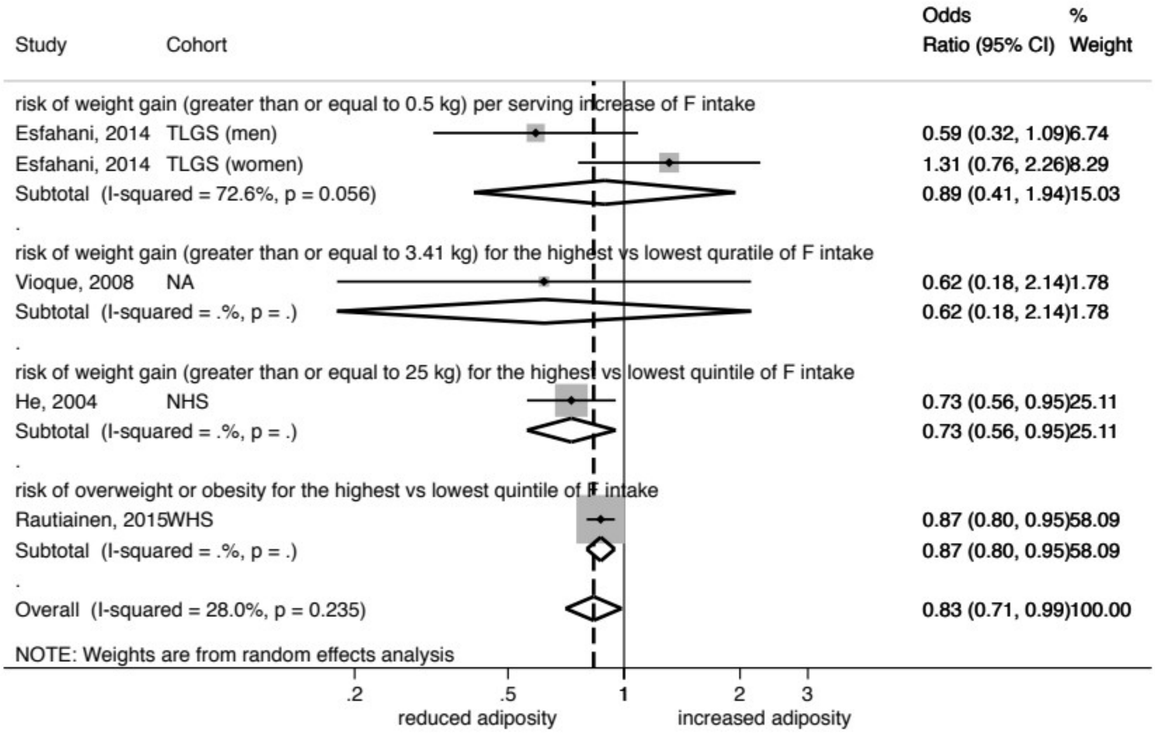
Study
Cohort
Odds Ratio (95% CI)
% Weight
risk of weight gain (greater than or equal to 0.5 kg) per serving increase of F intake
Esfahani, 2014 TLGS (men) 0.59 (0.32, 1.09) 6.74
Esfahani, 2014 TLGS (women) 1.31 (0.76, 2.26) 8.29
Subtotal (I-squared = 72.6%, p = 0.056) 0.89 (0.41, 1.94) 15.03
.
risk of weight gain (greater than or equal to 3.41 kg) for the highest vs lowest quratile of F intake
Vioque, 2008 NA 0.62 (0.18, 2.14) 1.78
Subtotal (I-squared = .%, p = .) 0.62 (0.18, 2.14) 1.78
.
risk of weight gain (greater than or equal to 25 kg) for the highest vs lowest quintile of F intake
He, 2004 NHS 0.73 (0.56, 0.95) 25.11
Subtotal (I-squared = .%, p = .) 0.73 (0.56, 0.95) 25.11
.
risk of overweight or obesity for the highest vs lowest quintile of F intake
Rautiainen, 2015 WHS 0.87 (0.80, 0.95) 58.09
Subtotal (I-squared = .%, p = .) 0.87 (0.80, 0.95) 58.09
.
Overall (I-squared = 28.0%, p = 0.235) 0.83 (0.71, 0.99) 100.00
NOTE: Weights are from random effects analysis
.2
.5
1
2
3
reduced adiposity
increased adiposity
